# Impact of losartan and angiotensin II on the expression of matrix metalloproteinase-9 and tissue inhibitor of metalloproteinase-1 in rat vascular smooth muscle cells

**DOI:** 10.3892/mmr.2014.2952

**Published:** 2014-11-14

**Authors:** YAN-SONG GUO, ZONG-GUI WU, JUN-KE YANG, XIN-JING CHEN

**Affiliations:** 1 Department of Cardiology, Provincial Clinical Medical College of Fujian Medical University, Fujian Institute of Cardiovascular Disease, Fujian Provincial Hospital, Fuzhou 350001, P.R. China; 2 Department of Cardiology, Changzheng Hospital, Second Military Medical University, Shanghai 200433, P.R. China; 3 Department of Cardiology, General Hospital of PLA Second Artillery, Beijing 100088, P.R. China

**Keywords:** losartan, angiotensin II, vascular smooth muscle cells, matrix metalloproteinase-9, tissue inhibitor of metalloproteinase-1

## Abstract

The present study aimed to investigate the impact of losartan and angiotensin II (AngII) on the expression of matrix metalloproteinase-9 (MMP-9) and tissue inhibitor of metalloproteinase-1 (TIMP-1), secreted by rat vascular smooth muscle cells (VSMCs). Rat VSMCs were isolated and cultured in different concentrations of AngII and losartan for 24 h and western blot analysis and quantitative polymerase chain reaction were performed to observe the subsequent impact on the gene and protein expression of MMP-9 and TIMP-1. AngII was shown to promote the protein and gene expression of MMP-9 in VSMCs in a concentration-dependent manner. No effect was observed on the expression of TIMP-1, therefore, an increase in the MMP-9/TIMP-1 ratio was observed. Losartan was shown to be able to inhibit MMP-9 protein and gene expression in a concentration-dependent manner, whilst promoting an increase in TIMP-1 expression, thus decreasing the ratio of MMP-9/TIMP-1. The combined action of losartan and AngII resulted in the same directional changes in MMP-9 and TIMP-1 expression as observed for losartan alone. The comparison of AngII, losartan and the combinatory effect on the expression of MMP-9 and TIMP-1 in VSMCs indicated that losartan inhibited the effects of AngII, therefore reducing the MMP-9/TIMP-1 ratio, which may contribute to the molecular mechanism of losartan in preventing atherosclerosis. In atherosclerosis, the development of the extracellular matrix of plaque is closely correlated with the evolution of AS. The balance between MMPs and TIMPs is important in maintaining the dynamic equilibrium between the ECM, and the renin-angiotensin-aldosterone system, which is involved in the pathologenesis of AS, and in which AngII has a central role.

## Introduction

Changes in the extracellular matrix (ECM) have been associated with numerous pathologies, including atherosclerosis (AS). Increased deposition of ECM follows various phases, including formation of fatty streaks and fibrous plaques, which can result in AS. A reduction in the ECM can result in the erosion and cracking of fibrous plaques, leading to myocardial infarction. The balance between the enzymes functioning to degrade the ECM and endogenous inhibitors is an important factor in determining the progression of AS and plaque stability (1). MMPs are the predominant enzymes which function to degrade the vascular ECM. The activation and proteolytic function of MMPs is regulated by tissue inhibitors of metalloproteinase-1 (TIMPs). Previous studies have suggested that MMP-9 and associated endogenous inhibitor TIMP-1 function across all stages of AS progression (1). Smooth muscle cells are the only cellular components of the arterial wall membrane of mammals. It has been previously confirmed that vascular smooth muscle cells (VSMCs) migrate from the arterial tunica media to the tunica intima, which results in phenotypic changes to the arterial wall membrane, and abundant proliferation and the formation of myogenic foam cells. This is important in the pathological development of AS (2,3). Newly derived VSMCs only have the capacity for binary fission, but secrete large amounts of ECM and active substances, including MMPs and TIMPs (4,5). Studying the impact of the various risk factors that promote the secretion of MMPs and TIMPs by VSMCs may be useful in the understanding of the pathogenesis of coronary heart disease. It is currently considered that the renin-angiotensin (Ang)-aldosterone system (RAAS) is involved in the pathological process of AS, in which AngII has a central part. Previous studies have suggested that losartan, an AngII receptor (AT1) antagonist, produces anti-arteriosclerosis effects. The present study therefore hypothesized that AngII and losartan may affect the secretion of MMPs and TIMPs by VSMCs, thus functioning in anti-AS or AS-induction (6–8). In the present study, rat VSMCs were cultured *in vitro* and analyzed for the effects of losartan and AngII in the secretion of MMP-9 and TIMP-1. The present study aimed to demonstrate the AS-induction effect of AngII and anti-AS effect of losartan.

## Materials and methods

### Primary cultivation of the adherent tissue blocks

The study was approved by the ethics committee of the Second Military Medical University (Shanghai, China). Male Wistar rats were obtained from the Animal Center of Shanghai Second Medical Military University (weight, 200–300 g; age, three-four months). They were fed with a standard diet and water, and housed at a temperature of 21–27°C. The thoracic aorta was surgically isolated from a healthy male Wistar rat. The adventitia and intima was removed, and the media layer was cut in tissue blocks sized ~1 mm^3^. The tissue blocks were transferred onto the walls of a 25 cm^2^ plastic culture flask, to which 5 ml Dulbecco’s modified Eagle’s medium (DMEM) with 20% newborn calf serum (NCS; Hangzhou Evergreen Company, Hangzhou, China) inactivated at 56°C for 0.5 h followed by packaging and preservation at 21°C (Hangzhou Evergreen Company), was added to the contralateral bottom. The flask was sealed and incubated at 37°C with 5% CO_2_ for 4 h. Following this, the culture flask was gently flipped for static cell culture. Following one week of culture, VSMCs were observed growing from the tissues, and after 2–3 weeks, a fused dense monolayer of proliferating cells formed. The cells were digested with 0.1% trypsin for passaging. The fourth to 10th generations of smooth muscle cells (SMCs) were obtained for subsequent experiments or frozen in liquid nitrogen.

### Cell synchronization

Following 3–4 days of subculture, synchronization was performed according to the requirements of the experiment. The supernatant was decanted, and the cells were washed with phosphate-buffered saline (PBS) 2–3 times. The cells were then added to the DMEM containing 0.5% NBS, which restrained the majority of cells to the G_0_ phase. When required, DMEM containing 20% NBS could be used to force the cells to proliferate (DNA synthesis phase).

### Identification of VSMCs

An inverted phase contrast microscope (CKX31-A12PHP; Olympus Corporation, Tokyo, Japan) was used to observe the morphology and growth patterns of living cells. Immunohistochemistry staining was used for detection of anti-α-actin as a specific indicator for VSMCs. Under sterile conditions, cover slips were used to cover the 6-well cell culture plates for VSMCs seeding. Following 48 hr of cultivation, the cover slips were removed and samples were washed three times for one min with PBS buffer, followed by fixation with 95% alcohol for 20 min. The streptavidin-peroxidase immunohistochemical method was then performed. This involved washing three times with PBS buffer, then soaking in 3% peroxidized methanol at room temperature for 20 min. Samples were then washed again three times with PBS buffer prior to addition of 50 μl 5% normal goat serum, followed by incubation at room temperature for 10 min. Mouse anti-rat SMA monoclonal antibody (50 μl, 1:500; Sigma) was added dropwise, and samples were then cultured at room temperature for 60 minutes. Following three washes with PBS, 50 μl biotinylated monoclonal goat anti-mouse immunoglobulin (Ig)G secondary antibody (1:200; Sigma) was added dropwise and samples were cultured at room temperature for 10 min. Sample were then washed three times with PBS, 50 μl streptomycin avidin-peroxidase solution was added followed by culturing at room temperature for 10 min. Freshly-configured DAB solution (100 μl) was added dropwise. Samples were then washed with water, restained with hematoxylin and mounted on neutral gum prior to observation under the microscope.

### Western blot analysis

The isolated cultured cells were seeded in culture plates at a concentration of 1×10^6^/ml. Following synchronization, the medium was replaced with DMEM containing 2% newborn calf serum (NCS). Different concentrations of losartan (Merck Co., Ltd., Whitehouse Station, NJ, USA) were added to the wells to produce final concentrations of 10^−7^, 10^−6^, 10^−5^ and 10^−4^ M. A well without addition of losartan was considered to be a blank control. The culture plate was incubated at 37°C and 5% CO_2_ for 24 h. The cells were then cultivated by collecting the supernatant and using a vacuum dryer to concentrate the samples. Coomassie blue staining method was used to determine the protein concentration. A protein sample of 20 μg was used for SDS-PAGE and western blotting. The procedure for western blotting was as follows. Thoracic aorta (50 mg)was cut into small pieces using a scalpel. PBS (1 ml) was added and tissues were homogenized twenty times using a glass homogenizer in an ice bath, followed by centrifugation at 2,124 × g at 4°C for 10 min. The supernatant was then collected and stored at −80°C. SDS-PAGE gel electrophoresis was used to separate the proteins. Samples were transferred on to a cellulose nitrate film (Hybond™-C; Amersham Biosciences, Piscataway, NJ, USA). Following transfer, the nitrocellulose membrane was placed into the closure solution (double distilled water) for 2 h. The closure solution was discarded and the membrane was washed three times with 0.1% (v/v) Tween-20 in Tris-buffered saline (TTBS; Biosharp, St. Louis, MO, USA) solution. TTBS was discarded and the primary mouse anti-rat MMP-9/TIMP-1 monoclonal antibody (diluted 1:500 with TBS) was added and incubated for 2 h. The primary antibody was then discarded and the membrane was rinsed three times with TTBS. TTBS was then discarded and the secondary alkaline phosphatase conjugated goat anti-mouse IgG monoclonal antibody (diluted 1:500 in TBS) was added and incubated for 2 h. The secondary antibody was then discarded, and the membrane was rinsed twice with TTBS and once with PBS. The appropriate amount of the staining reagents ) nitro-blue tetrazolium (NBT) and bromo chloro indole phosphate (BCIP), obtained from Sigma, were added, according to the manufacturer’s instructions and the colored bands were observed within 20 min. Distilled water was used to stop the reaction. The resultant bands were visualized with radiography and pictures were captured, scanned and analyzed using a computer image analyzer gel imaging system (GDS8000; Ultra-Violet Products, Upland, CA, USA). According to the instructions of the GDS8000 gel imaging system, the gray values of each signal band were then determined. Experiments were repeated four times, and the value of the control group was set as 1 for the calculation of each group’s relative value.

### Quantitative polymerase chain reaction (qPCR) and agarose gel electrophoresis

qPCR primers were synthesized by Shanghai Boya Biotechnology Co., Ltd. (Shanghai, China). The sequences were as follows: MMP-9 sense, 5′-GGCCTATTTCTGCCATGACAAATAC-3′ and antisense, 5′-CTGCACCGCTGAAGCAAAAG-3′; TIMP-1 sense, 5′-CCCCAGAAATCATCGAGAC-3′ and antisense, 5′-GATTATGCCAGGGAACCAG-3′; β-actin sense, 5′-GTGGGGCGCCCCAGGCACCA-3′ and antisense, 5′-CTCCTTAATTGTCACGCACGATTC-3′. Total RNA was extracted, followed using one-step RT-PCR kits obtained from Roche Diagnostics (Mannheim, Germany), according to the manufacturer’s instructions. The following conditions were used: 50°C for 60 min; 94°C for 30 min; 30 cycles of 94° for 30 sec, then 55°C for 30 sec, then 72° for 1 min; and 72°C for 7 min. A 10-μl sample of qPCR product and β-actin of the same sample were added to 4 μl of 6X bromophenol blue buffer. The samples were then separated by 1% agarose gel (0.5X TAE) electrophoresis at 100 V. After 1 h, nucleotides stained with bromophenol blue had migrated 3/4 of the total distance in the gel, and the electrophoresis was ended. The gels were imaged and a computer image analyzer scanned and analyzed the obtained bands. A comparison of the grayscale signals of each band were madde using a gel image analyzer (GDS8000) in order to semi-quantitively analyze the samples. This was repeated four times. The value of the control group was set as 1 for the subsequent calculation of the value of each group.

### Statistical analysis

The data are expressed as the mean ± standard deviation. An analysis of variance was performed where appropriate, and the Student–Newman–Keuls method was used for pairwise comparison. P<0.05 was considered to indicate a statistically significant difference.

## Results

### Identification of VSMCs

The SMC’s typical growth pattern was observed once the cells had grown and covered the bottom of the flask. The cells appeared spindle-shaped, growing in a parallel fashion, and arranged in cellular bunches. The dense and sparse areas overlapped and appeared in a “peak-valley” conformation.

Immunohistochemical staining used an antibody against smooth muscle actin (SMA; Sigma, St Louis, MO, USA). The cells were stained brown, giving positive confirmation that the cultured cells were VSMCs.

### Western blot analysis

**Figure 1:**
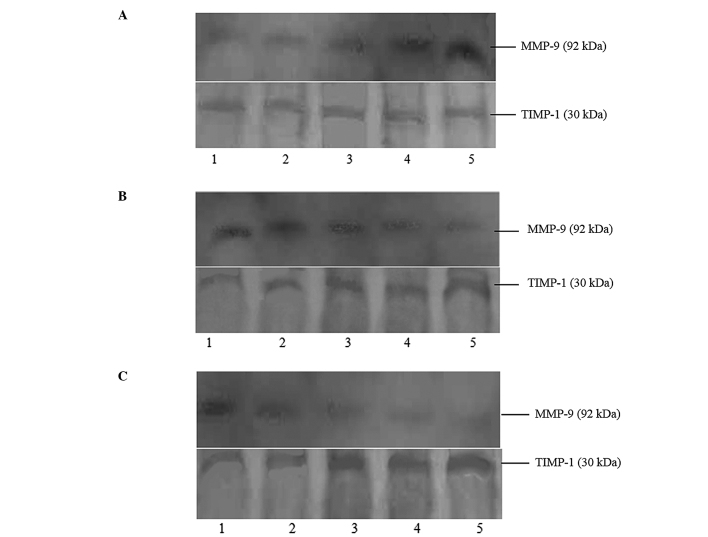
Effects of Ang II/losartan/losartan + AngII with different concentrations on expressions of MMP-9 and TIMP-1 protein in rat aortic vascular smooth muscle cells (24-h incubation). (A) Lanes 1–5 presented as blank, AngII 10^−9^ M, AngII 10^−8^ M, AngII 10^−7^ M and AngII 10^−6^ M, respectively. (B) Lanes 1–5 presented as blank, losartan 10^−7^ M, losartan 10^−6^ M, losartan 10^−5^ M and losartan 10^−4^ M, respectively. (C) Lane 1 presented as blank, and lanes 2–5 presented as added losartan 10^−7^ M, losartan 10^−6^ M, losartan 10^−5^ M and losartan 10^−4^ M based on AngII 10^−6^ M, respectively. MMP-9, matrix metalloproteinase-9; TIMP-1, tissue inhibitor of metalloproteinase-1; AngII, angiotensin II.

The effects of different AngII concentrations on the expression of MMP-9 and TIMP-1 protein in rat aortic VSMCs are shown in Fig. 1A. AngII could promote the expression of MMP-9, thus increasing the MMP-9/TIMP-1 ratio, in a concentration-dependent manner. AngII had no effect on the expression of TIMP-1 (Table I).

The effects of different concentrations of losartan on the expression of MMP-9 and TIMP-1 protein in rat aortic VSMCs are shown in Fig. 1B. Losartan inhibited the expression of MMP-9 and promoted the expression of TIMP-1. This resulted in a decrease in the MMP-9/TIMP-1 ratio in a concentration-dependent manner (Table II).

The effects of the combination of losartan and AngII on the expression of MMP-9 and TIMP-1 protein in rat aortic VSMCs are shown in Fig. 1C. The combination of AngII and losartan inhibited the expression of MMP-9 and promoted the expression of TIMP-1, therefore decreasing the MMP-9/TIMP-1 ratio, in a losartan-concentration-dependent manner (Table III).

### qPCR analysis

**Figure 2:**
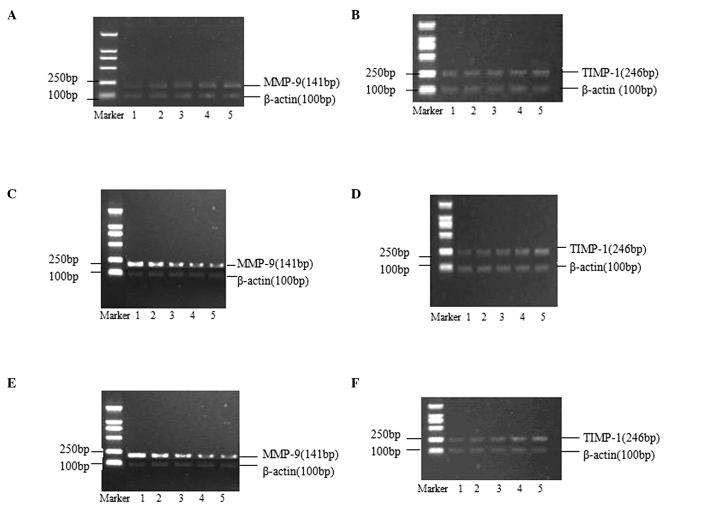
Effects of AngII/losartan/losartan + AngII with different concentrations on expressions of MMP-9 and TIMP-1 mRNA in rat aortic vascular smooth muscle cells (24-h incubation). (A and B) Lanes 1–5 presented as blank, AngII 10^−9^ M, AngII 10^−8^ M, AngII 10^−7^ M and AngII 10^−6^ M, respectively. (C and D) Lanes 1–5 presented as blank, losartan 10^−7^ M, losartan 10^−6^ M, losartan 10^−5^ M and losartan 10^−4^ M, respectively. (E and F) Lane 1 presented as blank, and lanes 2–5 presented added losartan 10^−7^ M, losartan 10^−6^ M, losartan 10^−5^ M and losartan 10^−4^ M based on AngII 10^−6^ M, respectively. MMP-9, matrix metalloproteinase-9; TIMP-1, tissue inhibitor of metalloproteinase-1. AngII, angiotensin II.

The effects of different AngII concentrations on the expression of MMP-9 and TIMP-1 mRNA in rat aortic VSMCs are shown in Fig. 2A and B. AngII could promote the expression of MMP-9 mRNA, increasing the MMP-9/TIMP-1 ratio, in a concentration-dependent manner. No effect of AngII was observed on the expression of TIMP-1 mRNA (Table IV).

The effects of different concentrations of losratan on the expression of MMP-9 and TIMP-1 mRNA in rat aortic VSMCs are shown in Fig. 2C and D. Losartan inhibited the expression of MMP-9 mRNA and promoted the expression of TIMP-1 mRNA, therefore decreasing the MMP-9/TIMP-1 ratio, in a concentration-dependent manner (Table V).

The effects of the combination of losartan and AngII on the expression of MMP-9 and TIMP-1 mRNA in rat aortic VSMCs are shown in Fig. 2E and F. The combination was observed to inhibit the expression of MMP-9 mRNA and to promote the expression of TIMP-1 mRNA, therefore decreasing the MMP-9/TIMP-1 ratio, in a losartan-concentration-dependent manner (Table VI).

## Discussion

The formation of AS is a slow process that may require decades in order to develop from initial fatty streaks to the advanced complex plaques. The progression of AS lesions is a consequence of three predominant processes. Firstly, damage to the arterial intima may cause lipid infiltration and deposition under the intima, accumulating cholesterol ester and free cholesterol in the cells and the surrounding connective tissue matrices. Secondly, a large number of VSMCs, macrophages and T lymphocytes may accumulate in the intima. Thirdly, VSMCs can migrate from the media to the intima, where they undergo large-scale proliferation, generating a large quantity of ECM and active substances. These biological processes are all closely associated with changes to the ECM. The ECM is an insoluble structural component that comprises the interstitial tissue and vascular matrix. The ECM has a physical and mechanical function in support of tissues and cells, as well as functioning to regulate the healing and fibration of tissue wounds, aging and cancer process of cells (9,10). The balance of the enzymes degrading the ECM and endogenous inhibitors are important in determining the progression of AS and plaque stability. During the development of AS, VSMC proliferation occurs across various periods of AS lesions, in the stage of fatty streak formation. The majority of cells are macrophages and macrophage-derived foam cells, together with a varying quantity of VSMCs. The number of VSMCs increases with the progression of AS, and becomes the major cellular component of the fibrous and atheromatous plaques. Previous research has shown that following endometrial injury, VSMCs migrate from the media to the intima and alter their form from a constriction to synthetic type (11). The synthesis and secretion of extracellular matrix components, including collagen, elastin and proteoglycan increase, together with changes in the secretion of active substances, including MMPs and TIMPs. As the only cellular component of the mammalian arterial blood wall, smooth muscle cells could not only secrete extracellular matrix, but additionally produce a variety of active substances, including enzymes which are able to degrade the ECM. Determining the changes in the levels of active substances secreted by VSMCs would have significant benefit in studies of AS.

It is currently considered that there are at least six major categories of ECM-degrading enzymes. These include prolidase, serine proteases, cysteine proteases, asparagine proteases, glycosidases, and MMPs, among which MMP is the most prominent. MMP exerts potent degradation effects on the ECM, thus acting as a central enzyme in the regulation of ECM homeostasis. MMPs may be divided into four different categories according to the target substrate. These include interstitial collagenase, gelatinase, matrical collagenase and membrane type metalloproteinases (12). The activation of progelatinase (MMP-9) on the cell surface predominantly functions in the degradation of the local matrix, which would be conclusive towards the migration and proliferation of cells. TIMPs are endogenous inhibitors of tissue MMPs, forming MMP-TIMP complexes at a ratio of 1:1, thus blocking the binding of MMPs and substrates and acting as a transcriptional regulation mechanism. TIMPs act to inhibit specific MMPs and it has been demonstrated that TIMP-1 specifically inhibits the activity of MMP-9 (13–15). The complex interactions of AS together with the release of growth factors can affect the MMP/TIMP balance, resulting in an increase in the MMP/TIMP ratio and an enhancement of the activities of collagenase, thus promoting the migration of VSMCs. Identifying the effects of the causative factors for the secretion of MMP-9 and TIMP-1 by VSMC may facilitate the understanding of the pathogenesis of coronary heart disease.

The RAAS system participates in the pathological process of AS, in particular AngII. AngII may act through the following hypothesized mechanisms: i) Induction of vasoconstriction, increased blood pressure, and consequently secondary induction of AS; ii) promotion of proliferation and vascular remodeling of the smooth muscle cells, inducing the mRNA expression and protein synthesis of type I and III collagen (16); iii) promotion of adhesion molecules, interleukin-6, monocyte chemotactic factor and other inflammatory cytokines. These three processes are mediated through the AT1 receptor. It is therefore presumed that AngII receptor antagonists have anti-atherosclerosis effects. Additional studies have suggested that losartan, an AT1 receptor antagonist, can produce an anti-atherosclerotic effect (17,18,7,8). This may occur through losartan-mediated inhibition of the degradation of partial matrices, which prevents the migration and proliferation of VSMCs from the media to the intima, and ultimately prevents the formation of AS plaques. The present study therefore analyzed the effects of losartan and AngII on the expression of MMP-9 and TIMP-1 secreted by rat VSMCs.

AngII has multiple functions in VSMCs, including affecting hemodynamics and cell growth, which can lead to the progression of ischemic coronary events. AngII is produced in the circulating blood or blood vessel walls, resulting in vasoconstriction and an increase in blood pressure. Production of AngII additionally has chronic effects, including the direct action on VSMCs, which leads to the remodeling of various cardiovascular tissues, including blood vessels and the heart (19). The impacts of AngII differ towards different cell types. In cultivation experiments of human and rat cardiac fibroblast cells, AngII was shown to stimulate the proliferation of cardiac fibroblasts, significantly decrease the collagenase secretion activity of cardiac fibroblasts, thus increasing the mRNA and protein expression of type I and III collagen. This process was shown to be mediated by the AT1 receptor (20). AngII was able to promote endothelial cell apoptosis in a dose-dependent manner through the combined mediation of AT1 and AT2 receptors (21) and was shown to inhibit VSMC apoptosis through the AT1 receptor (22). Co-incubation of cultured VSMCs with 1.7 nmol/l AngII promoted the secretion of type I collagen from the cells, which was reciprocally suppressed by losartan. Hadler-Olsen *et al* (23) reported that VSMCs derived from healthy individuals could secrete bioactive gelatinase in the culture medium; and this activity depended on the TIMP-bound proMMP, because it could not be fully activated by p-aminophenylmercuric acetate.

In the present study, analysis of the gene and protein expression levels showed that AngII could stimulate rat VSMCs to secrete MMP-9 in a dose-dependent manner, but had no effect on TIMP-1. Losartan was shown to inhibit the secretion of MMP-9 by rat VSMCs, at both the gene and protein levels, and promote the secretion of TIMP-1 in a concentration-dependent manner. The combined action of losartan and AngII could therefore inhibit the secretion of MMP-9 by rat VSMCs at the gene and protein level and promote the secretion of TIMP-1 in a concentration-dependent manner. The overall effect of losrartan with AngII was consistent with that of losartan alone. Morand-Contant *et al* (24) confirmed that AngII activates nuclear factor (NF)-κB in VSMCs through the AT1 receptor, Px. It was additionally shown that inhibition of NF-κB resulted in a decrease in expression of MMP-1, MMP-3 and MMP-9 in VSMCs (25). Another study showed (26) that AngII promoted the secretion of MMPs from human VSMCs through NF-κB signaling, which was blocked by losartan. The results of the present study were consistent with those of previous studies regarding the impact of AngII and losartan towards the secretion of MMP-9 by VSMCs. The occurrence and development of AS, as well as the rupture of plaques, are processes in which the balance of ECM-degrading enzymes and endogenous inhibitors is altered (1), however, in-depth studies of MMP action are required in order to determine the mechanism. It has been previously identified that TIMP-1 is the endogenous tissue inhibitor specific for MMP-9, functioning across the progressive stages of AS (1). The present study confirmed that AngII did not affect the secretion of TIMP-1 in VSMCs, while losartan could promote the secretion TIMP-1. It could therefore be concluded that AngII stimulated VSMCs to secrete MMP-9, altering the balance of MMP-9 and TIMP-1 to increase the MMP-9/TIMP-1 ratio. This observed trend towards an increase in collagenase activity, which promotes the migration and proliferation of VSMCs and other proinflammatory cytokines towards the intima and the formation of AS plaques and ultimate plaque rupture. Losartan could inhibit the AT1 receptor, altering the balance of MMP-9 and TIMP-1 secretion in VSMCs. The subsequent increase in the TIMP-1/MMP-9 ratio resulted in inhibition of collagenase activity, thus producing an anti-AS effect.

In conclusion, the present study showed that AngII could stimulate VSMCs to secret MMP-9 with no effect on TIMP-1 secretion. Losartan was shown to inhibit AngII via the AT1 receptor, promoting VSMCs to secret TIMP-1, thus inhibiting the secretion of MMP-9. Changes in the MMP-9/TIMP-1 secretion ratio in VSMCs may be one of the possible mechanisms for AngII promoting and losartan counteracting AS.

## Figures and Tables

**Table I:** Comparison of the gradation values of the obtained protein bands (Fig. 1A) as a result of different angiotensin II concentrations towards the expression of MMP-9 and TIMP-1 proteins in rat aortic vascular smooth muscle cells.

Groups	Control	10^−9^ M	10^−8^ M	10^−7^ M	10^−6^ M
MMP-9 gradation	1	1.06±0.08	1.35±0.11^a^	1.69±0.13^a^	2.22±0.18^a^
TIMP-1 gradation	1	0.98±0.08	1.05±0.11	0.98±0.10	1.04±0.12
MMP-9/TIMP-1	1	1.05±0.07	1.29±0.11^a^	1.72±0.13^a^	2.13±0.18^a^

a P<0.01 as compared with the control group. Values are presented as the mean ± standard deviation (n=4).

MMP-9, matrix metalloproteinase-9; TIMP-1, tissue inhibitor of metalloproteinase-1.

**Table II:** Comparison of the gradation values of the obtained protein bands (Fig. 1B) as a result of different losartan concentrations towards the expressions of MMP-9 and TIMP-1 proteins in rat aortic vascular smooth muscle cells.

Groups	Control	10^−7^ M	10^−6^ M	10^−5^ M	10^−4^ M
MMP-9 gradation	1	0.90±0.10	0.72±0.11^a^	0.58±0.13^a^	0.38±0.15^a^
TIMP-1 gradation	1	1.26±0.13^a^	1.63±0.13^a^	1.90±0.13^a^	2.19±0.13^a^
MMP-9/TIMP-1	1	0.71±0.06^a^	0.44±0.07^a^	0.31±0.05^a^	0.17±0.07^a^

a P<0.01 as compared with the control group. Values are presented as the mean ± standard deviation (n=4).

MMP-9, matrix metalloproteinase-9; TIMP-1, tissue inhibitor of metalloproteinase-1.

**Table III:** Comparison of the gradation values of the obtained protein bands (Fig. 1C) as a result of the combination of different losartan concentrations and angiotensin II towards the expressions of MMP-9 and TIMP-1 proteins in rat aortic vascular smooth muscle cells.

Groups	Control	10^−7^ M	10^−6^ M	10^−5^ M	10^−4^ M
MMP-9 gradation	1	0.93±0.07	0.7±0.06^a^	0.59±0.05^a^	0.37±0.04^a^
TIMP-1 gradation	1	1.28±0.09^a^	1.61±0.14^a^	1.88±0.17^a^	2.01±0.19^a^
MMP-9/TIMP-1	1	0.73±0.06^a^	0.43±0.04^a^	0.31±0.04^a^	0.18±0.02^a^

a P<0.01 as compared with the control group. Values are presented the mean ± standard deviation (n=4).

MMP-9, matrix metalloproteinase-9; TIMP-1, tissue inhibitor of metalloproteinase-1.

**Table IV:** Comparison of the gradation values of the obtained bands (Fig. 2A and B) as a result of different angiotensin II concentrations towards the expressions of MMP-9 mRNA and TIMP-1 mRNA in rat aortic vascular smooth muscle cells.

Groups	Control	10^−9^ M	10^−8^ M	10^−7^ M	10^−6^ M
MMP-9 gradation	1	1.28±0.11^a^	1.36±0.11^a^	1.58±0.10^a^	1.92±0.13^a^
TIMP-1 gradation	1	1.01±0.08	1.04±0.11	0.98±0.10	0.94±0.18
MMP-9/TIMP-1	1	1.27±0.04^a^	1.31±0.05^a^	1.61±0.06^a^	2.04±0.04^a^

a P<0.01 as compared with the control group. Values are presented as the mean ± standard deviation (n=4).

MMP-9, matrix metalloproteinase-9; TIMP-1, tissue inhibitor of metalloproteinase-1.

**Table V:** Comparison of the gradation values of the obtained bands (Fig. 2C and D) as a result of different losartan concentrations towards the expressions of MMP-9 mRNA and TIMP-1 mRNA in rat aortic vascular smooth muscle cells.

Groups	Control	10^−7^ M	10^−6^ M	10^−5^ M	10^−4^ M
MMP-9 gradation	1	0.91±0.10	0.69±0.11^a^	0.47±0.13^a^	0.31±0.15^a^
TIMP-1 gradation	1	1.26±0.13^a^	1.53±0.13^a^	1.80±0.13^a^	2.03±0.13^a^
MMP-9/TIMP-1	1	0.72±0.06^a^	0.45±0.07^a^	0.26±0.05^a^	0.15±0.07^a^

a P<0.01 as compared with the control group. Values are presented as the mean ± standard deviation (n=4).

MMP-9, matrix metalloproteinase-9; TIMP-1, tissue inhibitor of metalloproteinase-1.

**Table VI:** Comparison of the gradation values of the obtained bands (Fig. 2E and F) as a result of the combination of different losartan concentrations and angiotensin II towards the expressions of MMP-9 mRNA and TIMP-1 mRNA in rat aortic vascular smooth muscle cells.

Groups	Control	10^−7^ M	10^−6^ M	10^−5^ M	10^−4^ M
MMP-9 gradation	1	0.91±0.13	0.74±0.11^a^	0.59±0.10^a^	0.41±0.12^a^
TIMP-1 gradation	1	1.07±0.09	1.27±0.10^a^	1.77±0.12^a^	1.91±0.13^a^
MMP-9/TIMP-1	1	0.85±0.04	0.81±0.05^b^	0.76±0.06^a^	0.70±0.05^a^

a P<0.01 as compared with the control group,

b P<0.05 compared with the control group. Values are presented as the mean ± standard deviation (n=4).

MMP-9, matrix metalloproteinase-9; TIMP-1, tissue inhibitor of metalloproteinase-1.
